# Investigation of Polymer Adhesion of Materials in Multimaterial FFF Process

**DOI:** 10.3390/polym18070805

**Published:** 2026-03-26

**Authors:** Bálint Leon Seregi, Peter Ficzere, Gabriella Zsoldos

**Affiliations:** 1 Department of Railway Vehicles and Vehicle System Analysis, Faculty of Transportation Engineering and Vehicle Engineering, Budapest University of Technology and Economics, 1111 Budapest, Hungary; ficzere.peter@kjk.bme.hu; 2 Coloplast Hungary Ltd., 2800 Tatabánya, Hungary

**Keywords:** additive manufacturing, polymer adhesion, polymer compatibility, multi-material printing

## Abstract

The increasing availability of multi-material fused filament fabrication (FFF) systems has intensified the need for a systematic understanding of interfacial adhesion between model and support polymers. In this study, the adhesion behavior of commonly used engineering thermoplastics and dedicated support materials was investigated in the context of multimaterial FFF. A comprehensive experimental methodology was developed, including a custom tensile test specimen and fixture specifically designed to quantify interfacial adhesion under controlled conditions. Material combinations based on ABS, ASA, PETG, and carbon-fiber-reinforced PA (PAHT-CF), together with manufacturer-recommended and alternative support materials, were evaluated using uniaxial tensile testing and fracture surface analysis. The results demonstrate that interfacial adhesion strongly depends on material compatibility and processing conditions, and that dedicated support materials generally provide lower adhesion than model–model combinations. However, significant deviations were observed: SUPP PA exhibited unexpectedly high adhesion when paired with PAHT-CF, while SUPP ABS proved to be a more versatile support across multiple model materials, offering a favorable balance between sufficient adhesion during printing and ease of removal. Several material pairs showed negligible adhesion, leading to separation during manufacturing and limiting their practical applicability. Microscopic analysis revealed the coexistence of diffusion-driven bonding, mechanical interlocking, and weak boundary layer effects. The findings highlight that optimal support performance requires neither minimal nor excessive adhesion, and provide experimentally validated guidance for selecting material combinations and process windows in multimaterial FFF.

## 1. Introduction

With the development and rapid spread of additive manufacturing technologies, an increasing number of materials and more affordable equipment have become available. As a result, 3D-printed functional components are increasingly encountered as finished products or integrated elements. Among these technologies, material extrusion (Material Extrusion, MEX) is the most widely used. This is mainly due to its broad material portfolio and the fact that, compared to other additive manufacturing technologies, it is the easiest to use with low costs [1]. The fused filament fabrication technology (Fused Filament Fabrication, FFF) processes a thermoplastic polymer filament that is melted in an extrusion head and deposited in molten form through a moving nozzle, building up the desired geometry layer by layer using a CNC motion system. Due to its layered nature, the technology enables the production of almost any geometry, including internal components or complex internal channels with intricate paths. Although such geometries have been feasible before, their manufacturing using conventional technologies is significantly more expensive.

One of the main manufacturing constraints of the technology is the critical overhang angle, which is defined as the angle between a given surface and the build platform. Typically (depending on the material), surfaces with an angle lower than 45 degrees cannot be manufactured without additional support structures. These supports can be made either from the same material as the model or from a different material, depending on the machine configuration. When the support material differs from the model material, dedicated support materials are generally used, which cannot be employed independently as model materials due to their material properties. Two main types of such materials can be distinguished: breakaway and soluble supports. In the case of breakaway supports, easily accessible structures can be removed manually; their mechanical strength is sufficient only to support the printed mass and their own structure. Soluble support materials can also be used as breakaway supports; however, due to their solubility, they are significantly more expensive and are therefore applied only in cases where the support structure is inaccessible or difficult to reach, and where mechanical removal could damage the model [2,3]. The use of support structures has always represented a major limitation of the technology, as producing high-quality supported surfaces is costly, time-consuming, and requires experience to achieve proper settings. By applying the principles of Design for Additive Manufacturing (DfAM), the need for supports can be reduced; however, in certain cases, their use remains unavoidable. In recent years, multi-material printers have also become available at the consumer level. These solutions are implemented either by using multiple print heads or a dedicated filament feeding system. With such machines, the demand for improving the model–support interface has increased even further, as even manufacturer-recommended material combinations often fail to provide adequate process settings and surface quality [4,5]. In this work, this topic is investigated comprehensively, ranging from material characteristics through manufacturing parameters to the evaluation of the quality of the resulting surfaces. The goal of this study is to explore the adhesion between different widely used polymers in FFF/FDM technology with comparative analysis.

## 2. Polymer Adhesion

In multi-material additive manufacturing, the combined use of different filaments can be classified into three main functional purposes. One of the most widespread applications is the use of filaments made of the same base material but in different colors. This approach is most commonly applied for producing colored figurines, patterns, or inscriptions. In such cases, no post-processing is required to obtain multi-colored parts. For machine architectures that utilize a single nozzle, the flushing process during material changes is governed primarily by color rather than viscosity. For example, when switching from black to white filament, even a minimal residue of black material can cause visible discoloration in the printed part. To address this issue, various purge volume multipliers and specialized internal purge patterns are employed, which can be configured in slicing software.

Another key function of multi-material manufacturing is to enhance the performance of the manufactured component. Material selection in this case depends on the applied loads or operational conditions of the part. The objective is to combine materials that exhibit strong interfacial adhesion while exploiting specific properties of each component within the overall structure. For instance, if a component surface is required to be wear- and impact-resistant, while the overall part needs to remain relatively stiff, the materials of the outer shell and the internal infill structure can be tailored accordingly (e.g., HIPS for the outer walls and ASA for the internal structure). Related research also investigates solutions where material compatibility is not a primary concern; instead, mechanical bonding is achieved through interlocking lattice structures embedding the different materials within each other [6,7]. Beyond lattice-based solutions, the performance of additional bonding geometries is also being studied through model-level and slicer-level modifications [8,9].

**Figure 1:**
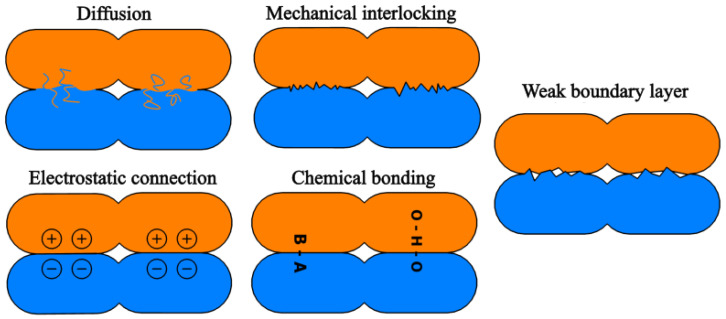
Types of adhesions between polymers [10,11].

In the case of additively manufactured parts, adhesion mechanisms can be evaluated through interlayer bonding behavior using tensile stress measurements and fracture surface analysis. Adhesion strength is governed simultaneously by multiple adhesion mechanisms (Figure 1) [10,11]:

- Diffusion adhesion: During neck formation, polymer chains penetrate into the cross-section of the adjacent layer across the interface. A local material bridge (neck) forms at the contacting surfaces of the filaments (Figure 1). An increased neck size enhances material continuity and promotes viscous flow between adjacent filaments. This adhesion mechanism is strongly influenced by temperature, molecular chain length, and polymer mobility. Elevated nozzle and chamber temperatures combined with low printing speeds (i.e., prolonged thermal exposure) facilitate polymer chain diffusion. During cooling, improved chain entanglement and larger neck cross-sections contribute to increased bond strength.
- Mechanical adhesion: This mechanism arises from form closure caused by surface roughness and defects such as micro-grooves. The freshly deposited, lower-viscosity melt flows into surface irregularities and solidifies, forming a mechanical interlock. Optimal bonding is achieved when the previously deposited layer has partially solidified, while the newly deposited layer exhibits sufficient wetting behavior. This adhesion type is influenced by the viscosities of the two materials, as well as nozzle and chamber temperatures.
- Electrostatic adhesion: Although not directly measurable, this mechanism is theoretically recognized. An electrical double layer forms between the contacting materials, generating Coulombic attraction forces due to charge displacement at the interfaces. In FFF processes, the materials used are typically electrically insulating; therefore, the contribution of electrostatic adhesion to the overall bonding strength is generally negligible.
- Chemical adhesion: Chemical bonding may form at the interface between the contacting materials. Depending on the available functional groups and molecular structures at the surfaces, covalent, ionic, or hydrogen bonds may develop. Among the adhesion mechanisms, chemical bonding is considered the strongest. In polymer-based FFF systems, hydrogen bonding is most likely to occur, particularly when the polymers contain highly electronegative atoms. Covalent bonds typically do not form due to the temperature range applied during printing.
- Weak boundary layer: This is a conceptual model that accounts for surface contamination, trapped air, and other defects that reduce the effective contact area. It influences all adhesion mechanisms and has a significant impact on the measurable bond strength.

In multimaterial FFF, interfacial adhesion should not be interpreted as a single phenomenon, but rather as a sequence of coupled events. After deposition, the newly extruded strand first has to establish intimate contact with the previously deposited material. In practice, this stage is governed by the combined effect of extrusion pressure, local wetting, melt viscosity and surface topography. Once sufficient contact is achieved, a neck develops between the adjacent filaments, increasing the real load-bearing area of the interface. Strength development then proceeds through interdiffusion of polymer chains across the interface and their subsequent re-entanglement. For thermoplastic polymers, this healing process is strongly time- and temperature-dependent, and under FFF conditions it remains inherently non-isothermal because the interface cools rapidly after deposition. As a consequence, higher nozzle temperature, lower printing speed, smaller layer height and longer local thermal exposure generally promote stronger bonding, provided that thermal degradation does not occur [12,13,14,15].

For dissimilar polymers, the above sequence is further constrained by thermodynamic compatibility between the two materials. Recent work on multi-material extrusion has shown that smaller differences in Hansen solubility parameters and coefficient of thermal expansion are associated with higher interface strength, indicating that wettability, physical adsorption, intermolecular diffusion and thermal stress development all contribute to the final bond quality. In semi-crystalline systems, crystallization competes directly with diffusion: as the material crystallizes, chain mobility decreases, and the healing process may be arrested before a strong interpenetrating interface is formed. This aspect is particularly important for polyamide-based systems and for fiber-reinforced materials, where the filler phase can increase melt viscosity, reduce the effective polymer-polymer contact area and shorten the time available for diffusion bonding. Moreover, voids, trapped air and incomplete local fusion act as a weak boundary layer, reducing the effective contact area and promoting premature interfacial failure. Accordingly, the experimentally measured adhesion in multimaterial FFF should be understood as the resultant of intimate contact formation, wetting, chain diffusion, material compatibility and defect population, rather than as the manifestation of a single isolated mechanism. This also explains why different material pairs may show similar nominal processing temperatures but substantially different interfacial strengths [16,17].

In practice, individual adhesion mechanisms are difficult to separate experimentally. For example, increasing the temperature enhances diffusion but simultaneously improves mechanical adhesion due to better melt wetting. Therefore, rather than isolating individual adhesion mechanisms, the resultant adhesion governed by a given set of manufacturing parameters is the most relevant and practically measurable quantity.

## 3. Measurement of Polymer Properties

### 3.1. Tensile Test

**Figure 2:**
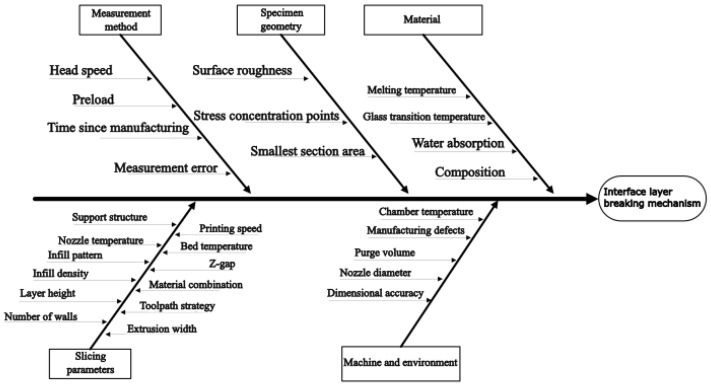
Potential factors influencing the adhesion and breaking mechanism between two polymers regarding FDM/FFF materials based on [23].

The tensile properties of polymers are measured in accordance with the MSZ EN ISO 527-1 and ASTM D638 standards [18,19]. These standards do not specifically address the testing of additively manufactured parts; however, in the absence of dedicated standards, they are commonly adopted as a reference framework by researchers [20]. Experimental studies have highlighted that, in addition to the inherently anisotropic nature of the technology, both manufacturing and testing parameters have a significant influence on the measured results [21,22]. Figure 2 summarizes the potential influencing factors that may affect the fracture mode of an FFF-printed test specimen. Factors ranging from the manufacturing environment and raw material characteristics to the applied testing methodology must be considered, as they can influence the results in various ways [23]. Certain parameters, such as chamber temperature and build plate temperature, are interdependent; however, understanding these cause–effect relationships is essential for robust design and reliable interpretation of experimental results.

#### 3.1.1. Effect of Testing Speed

The tensile testing speed has a significant influence on the measured results when testing polymer-based specimens. As the testing speed increases, the tensile response exhibits a more brittle behavior. Increasing the crosshead speed affects the Young’s modulus, and noticeable differences can also be observed on the fracture surfaces. Zrida et al. investigated polypropylene specimens at strain rates of 8, 25, and 200 s^−1^. The results showed that fracture surfaces observed at a strain rate of 8 s^−1^ exhibited a relatively smooth appearance with only a few cavities. At higher strain rates, the fracture surfaces became rougher, and a large number of depressions appeared. When the strain rate reached 200 s^−1^, several white, spherical features began to form as a result of localized overheating [24].

#### 3.1.2. Effect of Temperature

In addition to the crosshead speed, the ambient temperature and consequently the temperature of the test specimen also have a significant influence on the measurement results. Higher temperatures drastically reduce tensile strength and elastic modulus. This behavior is primarily related to the distance from the glass transition temperature. At lower temperatures, the measured response tends to be more brittle, whereas contraction and larger deformations are observed at higher temperatures [25]. Therefore, not only during mechanical testing but also in practical applications, it is essential to consider the expected operating temperature of the printed component in order to select an appropriate material accordingly.

#### 3.1.3. Effect of Manufacturing Parameters

Mechanical properties of additively manufactured components can also be tailored through manufacturing parameters. Focusing specifically on FFF technology, layer height, printing temperature, build orientation, and the internal infill pattern have a strong influence on strength and stiffness. Certain manufacturing parameters such as chamber temperature, build plate temperature, and nozzle temperature are primarily material-dependent and must be selected to achieve optimal melt viscosity and wetting behavior. These effects are most prominently reflected in porosity levels and interlayer bonding quality. Other parameters, such as infill pattern and infill density, influence stiffness from a topological perspective due to their effect on the effective cross-sectional properties of the printed part [26]. Thermal manufacturing parameters are particularly relevant for improving interlayer properties, as they directly affect bonding mechanisms. Increasing the nozzle temperature enhances bonding; however, excessively high temperatures may lead to dimensional inaccuracies due to excessive material flow. Heating the part from below via the build plate promotes molecular diffusion, although similar effects can also be achieved by reducing the printing speed, thereby increasing thermal exposure from the radiative heat of the nozzle [27]. An additional challenge arises from improper selection of printing speed, as it has a direct impact on tensile strength. This effect is caused not only by thermal treatment due to nozzle radiation but also by changes in volumetric flow rate. Each print head has a maximum volumetric flow rate, which defines the maximum material throughput that can be achieved without under-extrusion. This limit can be slightly increased by raising the nozzle temperature; however, the material itself imposes an upper limit to avoid reaching its degradation temperature. Consequently, even if a printer is capable of high motion speeds, the print head may not be able to sufficiently heat the material across the full cross-section of the melt zone, thus limiting the achievable volumetric flow rate [28].

## 4. FFF Model–Support Interface

Surfaces with overhang angles below the critical threshold require support structures, as the extruded filaments are largely deposited in mid-air and tend to sag during extrusion. As a result, the external surface or even the entire geometric feature may exhibit significant dimensional inaccuracies [29]. In machines where automatic material switching within the extruder is not available, the support material and the model material are identical. When the support structure is fabricated from the same material as the model, special slicing settings must be applied to the support in order to prevent complete fusion between the two [30]. In printers equipped with multiple extruders or automatic material-changing systems, the support material may differ from the model material. In such cases, material combinations must be selected that do not fuse or only partially fuse during printing.

**Figure 3:**
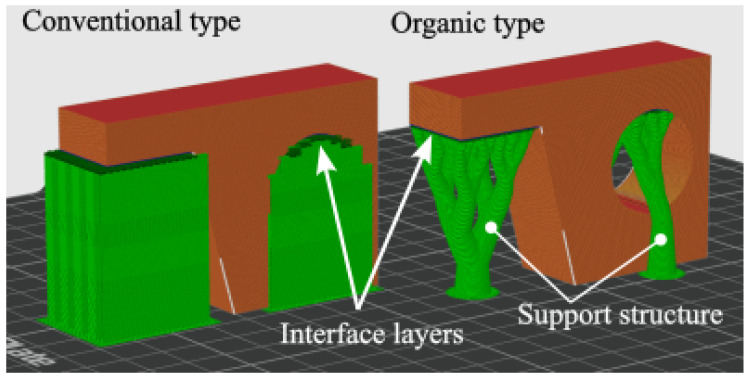
Types of support structures generally used for FDM/FFF printing.

In general, two main types of support structures can be distinguished (see Figure 3). The first is a solid, infill-based structure, while the second is an organic, so-called tree-type structure. For both types, it can be specified whether the supports are generated only directly beneath the overhanging surfaces (and may therefore be built on top of the model itself) or whether they must always make contact with the build platform. Tree-type supports require significantly less material and shorter build times, whereas conventional solid supports provide greater stiffness and more reliable mechanical support [31].

Insufficient adhesion between the model and the support structure may also lead to printing failures. When a model feature does not directly contact the build platform, it is supported solely by the support structure. During printing, lateral forces arising from nozzle interaction can cause the model to shift or detach from the support. In certain cases, this may result in print head collisions and subsequent machine shutdowns. Additional issues may arise due to differences in the coefficients of thermal expansion between the model and support materials. In large parts, where the chamber temperature is insufficiently high, warping-induced deformation can lead to separation between the model and the support structure.

## 5. Methodology

After completing the literature review and defining the research objectives, the development of the methodology was carried out. The problem was approached primarily from a practical perspective in order to select parameters and parameter combinations that would have a direct impact on the results and reflect real industrial manufacturing practice. Prior to finalizing the experimental design, the development of the specimen geometry, the gripping fixture, and the investigated material combinations was conducted in parallel. The design process started with the gripping fixture required for the testing machine, followed by the specimen geometry adapted to this fixture. During material selection, commonly used engineering polymers from the same manufacturer were chosen. Subsequently, the experimental plan was established using a combination matrix, and preliminary measurements were performed on a limited number of specimens to verify the reliability of the testing procedure. In the case of identified issues (discussed later with respect to the measurement range and specimen geometry), the measurement configuration was revisited and adjusted. After successful preliminary testing, large-volume specimen production was scheduled, followed by staged measurements and subsequent data evaluation.

### 5.1. Material Set

The experimental investigation was conducted exclusively using materials commercially available from Bambu Lab. Specifically, engineering-grade polymers and their corresponding recommended support materials were selected. Information regarding material properties and recommended processing parameters was obtained from technical data sheets and safety data sheets (Table A1). Based on the collected data, feasible material combinations could be identified more easily, as multiple parameters significantly influence printability. Care was taken to ensure that all test specimens (5 pieces per combination) were manufactured from the same filament spool for each material.

### 5.2. Variations

Since freedom in parameter adjustment is crucial in experimental design, the specimens were manufactured using a Bambu Lab X1C printer (Bambu Lab, Shenzen, China) instead of an industrial machine that would limit the printing parameter options. Although this system is capable of automatic material switching within the print head, the use of a single nozzle requires a purge extrusion during material changes. This introduces a limitation: when purging a material with a higher melting temperature using a material with a lower melting temperature, nozzle clogging may occur. Under elevated temperatures, the incoming filament may soften excessively, preventing proper feeding by the extruder gears, or the more viscous material may not be fully purged from the melt zone of the hotend. To mitigate this issue, the recommended printing temperatures of the investigated materials were collected (Table 1), and their temperature differences were summarized in a combination matrix. A maximum allowable temperature difference of 25 °C was defined, and feasible material combinations were filtered accordingly (Table 2). As shown in Table 1, the default slicer profiles typically recommend the upper limit of the temperature range specified in the material data sheets [32,33,34,35,36,37]. This is likely due to the high printing speeds used, as achieving a fully molten state across the entire filament cross-section is more challenging at high volumetric flow rates within the relatively small melt zone of the hotend. Insufficient melting may lead to extrusion issues and poor interlayer adhesion in the printed parts.

### 5.3. Measurement Configuration

**Figure 4:**
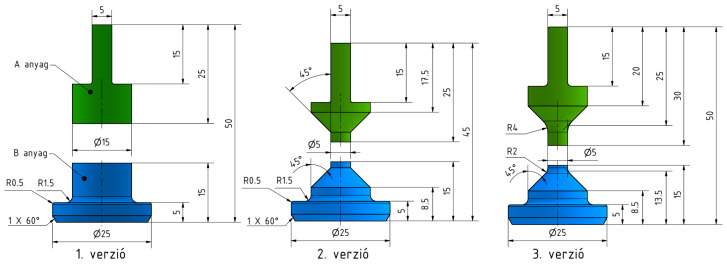
Versions of the specimen geometry.

The objective of the measurement was to evaluate the adhesion strength between the two material components. The test specimen was subjected to uniaxial tensile loading in a universal testing machine, where displacement and applied force were recorded. A custom specimen geometry was designed to enable measurement on the available testing equipment (Figure 4, Version 1).

**Figure 5:**
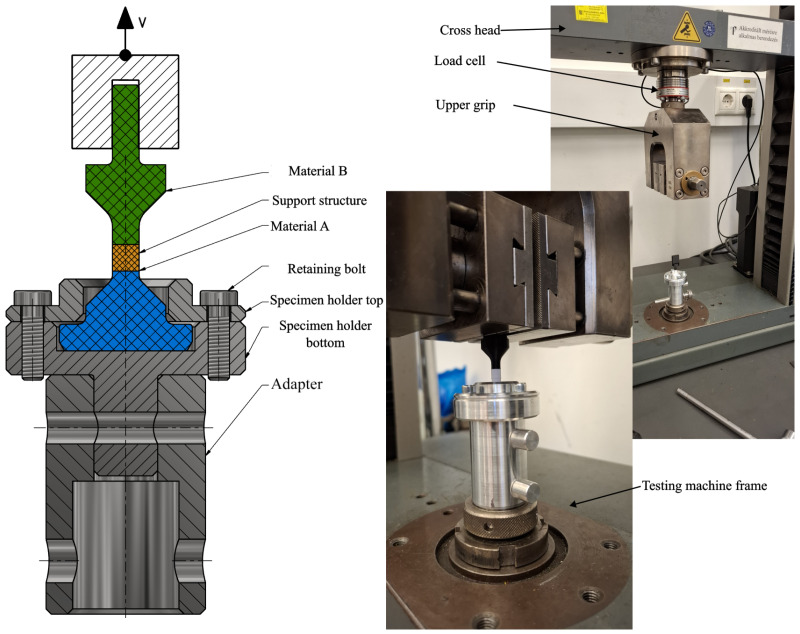
Configuration of the measurements.

A dedicated fixture compatible with the testing machine grips was also designed to match the specimen geometry (Figure 4), allowing rapid test setup and accurate specimen alignment. Preliminary tests were conducted using an Instron 5543 universal testing machine equipped with a 50 N load cell (Figure 5). The initial specimen geometry required forces exceeding the load cell capacity, resulting in automatic shutdown. Consequently, the specimen cross-section was reduced at the material interface (Figure 4, Version 2), inspired by geometries presented in [38,39,40], while maintaining a straight interface between the two materials. Despite this modification, the load cell was still overloaded, prompting further optimization through slicing parameter adjustments and another testing machine was used for measurements (Zwick Z005T with a 1 kN load cell).

### 5.4. Slicing Parameters

For the purposes of the measurement, it was essential that only the material varied between test cases. To ensure comparability of the results, a parameter set was defined that satisfied the processing requirements of all materials. Special attention was given to the support structure settings to ensure sufficient strength, promoting failure at the material interface rather than within one of the bulk materials. It should be noted that such support configurations are not representative of standard industrial practice and were selected solely to ensure reliable measurement. The customized slicing parameters are summarized in Table 3. All other parameters not explicitly listed were kept at their default values in the Bambu Studio X1C profile for a 0.4 mm nozzle and 0.2 mm layer height (version 1.7.1.62).

#### 5.4.1. Designation System

**Figure 6:**
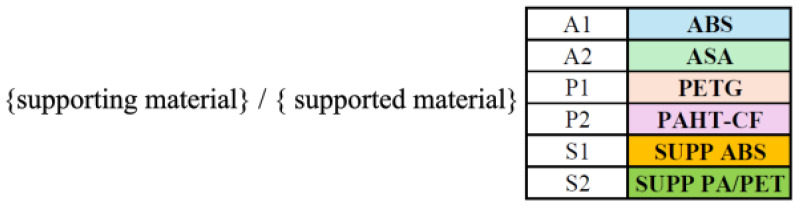
Structure of the specimen identification codes and notation of the investigated materials.

The test specimens were manufactured in batches by material combination within a single print job (5 specimens per program), thereby ensuring consistent environmental conditions. Each print job consisted of five specimens, and the numbering also indicates the production order within the job. The structure of the specimen identification code and the abbreviated notation of the materials are illustrated in Figure 6.

#### 5.4.2. Result Evaluation

To evaluate the fracture stresses, the diameter of the specimen was measured via digital calipers at the material interface ($D_{fracture}$). From this measurement, the section areas were calculated ($A_{fracture}$). Dividing the maximum measured forces ($F_{max}$) by these area values, the fracture stresses are defined:

(1) $$\sigma_{max}=F_{max}/A_{fracture}$$

Fractures that occurred inside the model or support part instead of the interface were not considered in the statistics. The deviations of the results were presented accordingly.

## 6. Results

### Tensile Stresses

**Figure 7:**
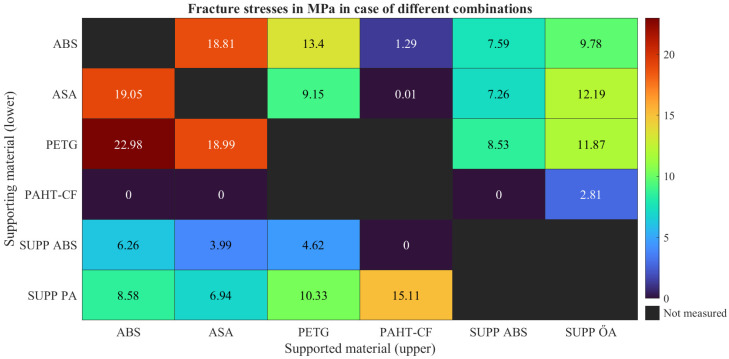
Fracture stresses measured in case of all the investigated polymer combinations.

**Figure 8:**
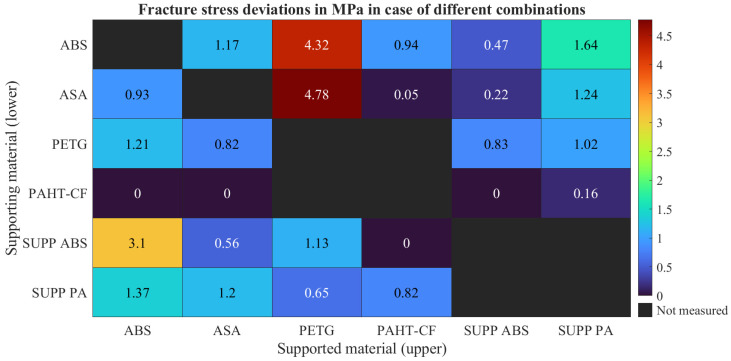
Fracture stress deviations measured in case of all the investigated polymer combinations.

The fracture stresses were calculated from the maximum tensile forces and the measured specimen diameters. The obtained values are summarized in Figure 7, while the corresponding standard deviations are shown in Figure 8.

For all investigated material combinations, the standard deviation values remained within the range typically reported for tensile strength data in material datasheets. Several material combinations resulted in measurable fracture stresses, whereas others showed no measurable adhesion. In these cases, separation occurred during manufacturing, storage, or specimen mounting, resulting in zero values in the evaluation.

The black, unannotated fields indicate material combinations that were not manufactured or tested. Identical material pairings without an interface gap were excluded, since such configurations would lead to excessively high adhesion and are not relevant for practical support applications. The PAHT-CF/PETG combination was also excluded from fabrication because of the large difference in processing temperatures and the associated risk of nozzle clogging.

## 7. Discussion

### 7.1. Supported ABS

**Figure 9:**
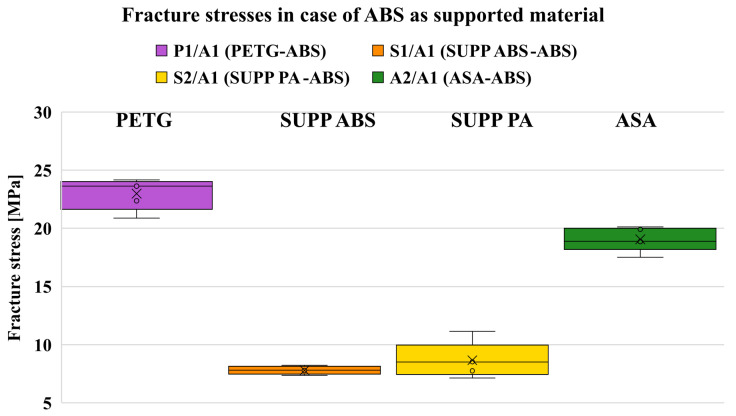
Fracture stresses of different combinations when supporting ABS.

Grouping the results by model material allows direct comparison of the corresponding support materials. In the case of ABS, the relevant support materials are PETG, ASA, SUPP ABS, and SUPP PA. As shown in Figure 9, the two model materials exhibit significantly higher fracture stresses compared to the support materials. The support material specifically recommended for ABS shows higher adhesion than SUPP PA, which is initially surprising; if SUPP PA were equally suitable for supporting this material, the question arises why two different support materials are offered. The answer can be found in Figure A5. Observing the supported surfaces, it is evident that SUPP ABS yields a much more uniform, flatter surface than SUPP PA. This indicates that the contacting surfaces contain fewer defects and that melt flow and wetting behavior are more favorable within this temperature range. Consequently, when selecting a model–support material combination, it is important to define what is expected from the interface: in some applications, a compromise must be made between achieving a high-quality supported surface and maintaining low adhesion. In case of too much adhesion, it poses a risk of failure by the dissimilar model materials sliding on the support.

**Figure 10:**
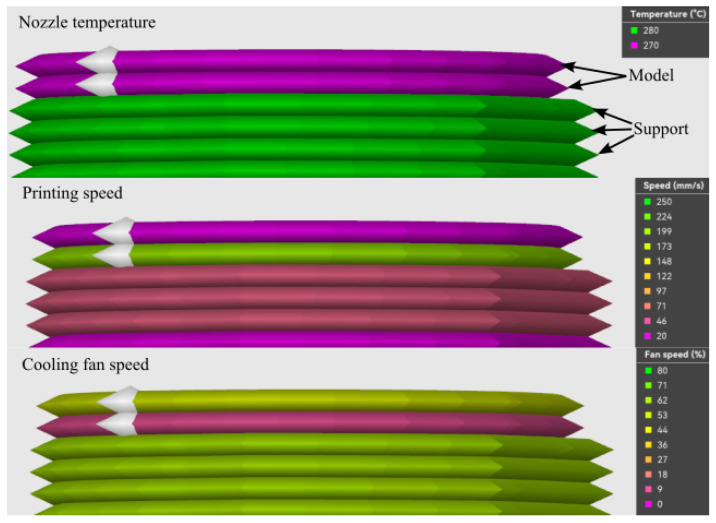
Higher printing speed of the initial model layer as shown in the slicer [41].

**Figure A1:**
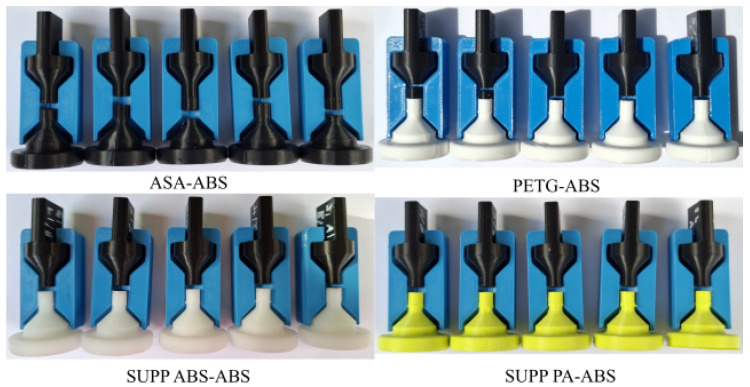
Fracture locations for ABA model material.

Examining the specimens (Figure A1), it can be observed that fracture did not always occur exactly at the material interface. For combinations with ASA and PETG, the fracture location shows considerable scatter; however, across all combinations, a clear tendency appears: near the interface, one or two layers consistently detach from the model material side. This failure mode indicates that the interlayer adhesion between the first two printed model layers is weaker than the adhesion at the interface between the two dissimilar materials. Inspecting the default slicer settings and toolpaths, it becomes evident that the first supported model layer is printed with parameters differing substantially from those of the subsequent layers. Figure 10 shows the toolpaths of the last support layers and the first two model layers printed on top of the support, colored according to different parameter values. It is clearly visible that, although the nozzle temperature remains unchanged for the first two model layers, the printing speed is significantly higher and the part cooling fan speed is lower than for the subsequent layer. The cooling reduction for the first model layer can be important for maximizing the contact area between the two materials. The cooling rate and printing speed difference cause a weaker bond between the first two layers of the model material side. This explains why one or two layers often remain attached to the support structure after fracture and, in line with the observations reported in [41], suggests that in this case the goal is to reduce, rather than increase, adhesion. Finally, supporting PETG can be compared to the literature, where the tensile stresses were slightly higher (29.3 +/− 0.8 MPa) than the result in this work. In this literature, the measurement speed was lower (2 mm/min instead of 5 mm/min) and the used nozzle diameter was 0.8 mm, which is twice the size of the nozzle used in this work [42].

Microscopic examination of the fracture surfaces also provides insight into the governing adhesion mechanisms. On the left side of Figure A6, dark discoloration is visible on the PETG surface, indicating diffusion, while on the right side, PETG embedded into ABS is visible as a result of mechanical interlocking. For the ASA combination, no clear interface could be identified on the fracture surface, as the specimens failed within the bulk material; moreover, this was the only combination where fracture occurred within the support structure itself (Figure A7). The fracture surfaces of SUPP ABS and SUPP PA have already been shown in Figure A5. On these surfaces, no evidence of diffusion can be observed, suggesting that the bond strength is primarily governed by the weak boundary layer and mechanical adhesion. The measurement results of the combinations, including corrected true diameter, fracture force, and fracture stress, are summarized in Table 4.

#### 7.1.1. Supported ASA

**Figure 11:**
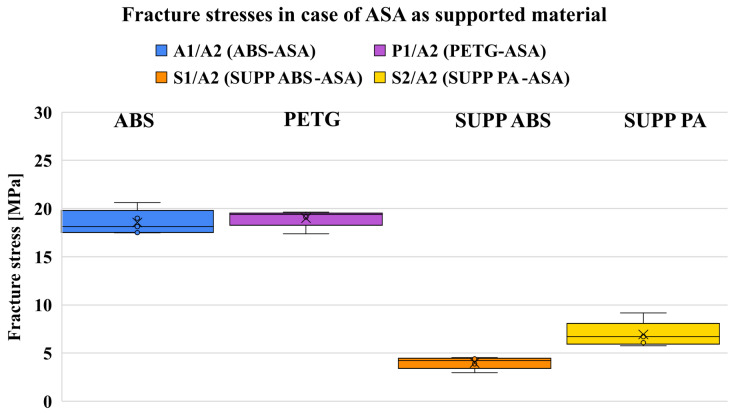
Fracture stresses of different combinations when supporting ASA.

For ASA as model material, Figure 11 shows that the two dedicated support materials provide the lowest adhesion. The standard deviation values are low for all measured combinations, with the highest value being only 1.2 MPa. ABS and PETG exhibit similarly higher adhesion levels compared to the combinations using SUPP ABS and SUPP PA.

**Figure A2:**
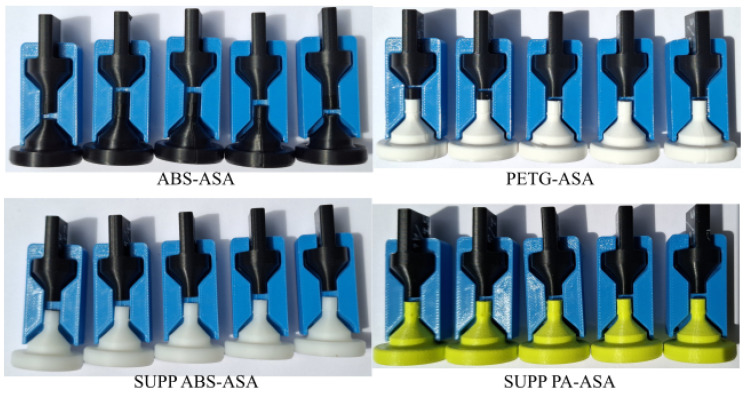
Fracture locations for ASA model material.

Examining the fracture surfaces, it can be stated that the dedicated support materials provide an appropriate bond to ASA, as they offer sufficient adhesion to prevent the model from detaching during printing, yet fractures consistently occurred at the interface under uniaxial tension at relatively low stresses (Figure A2). For the combinations printed with ABS, the specimens fractured within the bulk material, predominantly in the neck region or near the boundaries of the support structure, in accordance with the weak boundary layer concept. This indicates that this combination is not suitable as a support pair. A similar conclusion can be drawn for PETG, although one specimen did fail at the interface. Microscopic examination of the interfacial fracture surface shows strong diffusion bonding between the materials, manifested as visible discoloration (Figure A9). Beyond diffusion, the weak boundary layer concept again appears to play a dominant role, as the deposited model material exhibits good spreading and thus a large real contact area. Compared to the literature, the PETG/ASA combination showed more than two times higher stress (18.99 MPa compared to 8.8 MPa). In the reference work, the standard ISO 527 dogbone geometry was used, but there is no sufficient data regarding the measurement parameters [43]. For SUPP ABS and SUPP PA, the observations are similar to those made for ABS as a model material. SUPP ABS provides a smoother supported surface than SUPP PA; however, in terms of fracture stress values, these support materials exhibit even lower adhesion to ASA than to ABS. The averaged measurement results are presented in Table 5.

#### 7.1.2. Supported PETG

**Figure 12:**
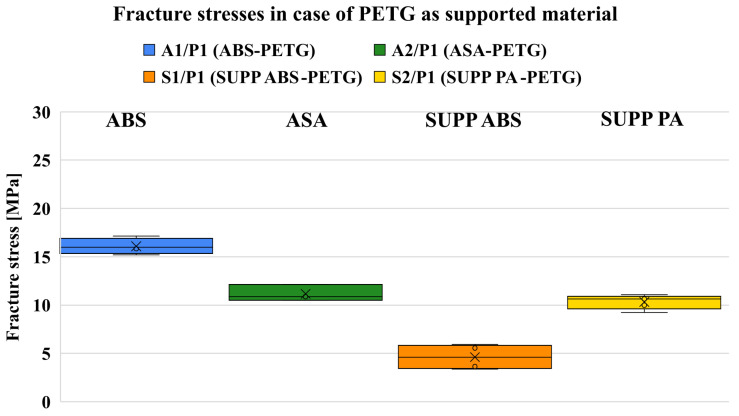
Fracture stresses of different combinations when supporting PETG.

**Figure 13:**
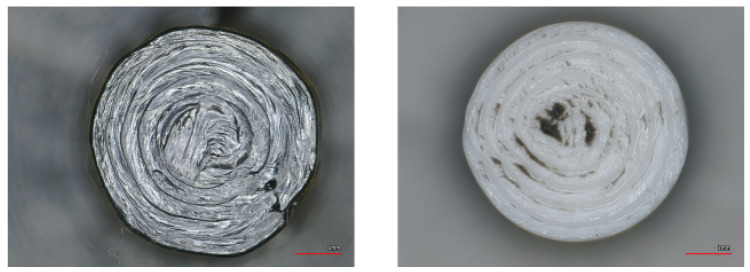
Diffusion at the interface between ASA and PETG. Reference length: 1 mm.

**Figure A3:**
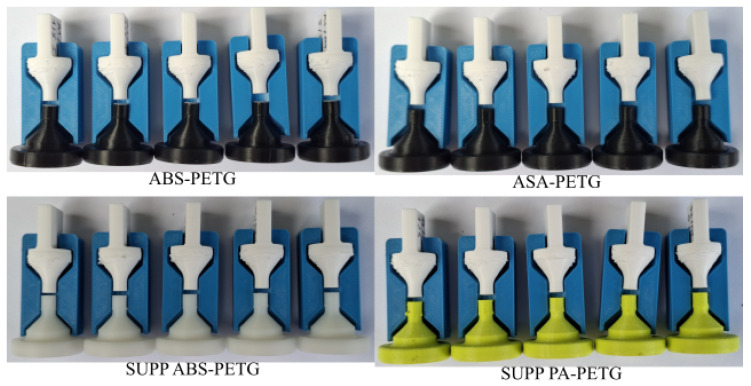
Fracture locations for PETG model material.

For PETG as model material, SUPP ABS proved to be the most effective support, followed closely by ASA and SUPP PA, while ABS exhibited the highest fracture stress (Figure 12). However, these values are somewhat misleading, as the PETG regions of the specimens show poor surface quality (Figure A3). The deterioration in quality is not caused by insufficient purge volume, as no material transition or discoloration is visible on the parts (only diffusion is present, as seen in Figure 13). Although higher printing and chamber temperatures increase the degree of diffusion [44,45], the primary source of error here is the chamber temperature. Among the materials investigated, PETG requires the lowest nozzle and build plate temperatures. During manufacturing, chamber temperatures were also recorded. For print jobs where PETG was used as support material (i.e., with a build plate temperature of 70 °C), an average chamber temperature of around 40 °C was measured. In contrast, when PETG was used as model material, the chamber temperature was 5–10 °C higher, which proved to be excessive. This is understandable, considering that the glass transition temperature of PETG is 68 °C, and in practice, chamber heating is typically not used when printing with PETG. Overall, it can be concluded that the investigated support material combinations are not recommended for PETG as a model material. In practice, PLA is most commonly used as a support for PETG, as this combination shows low adhesion and their printing parameters do not differ significantly [46]. The averaged measurement results are summarized in Table 6.

#### 7.1.3. Supported PAHT-CF

**Figure 14:**
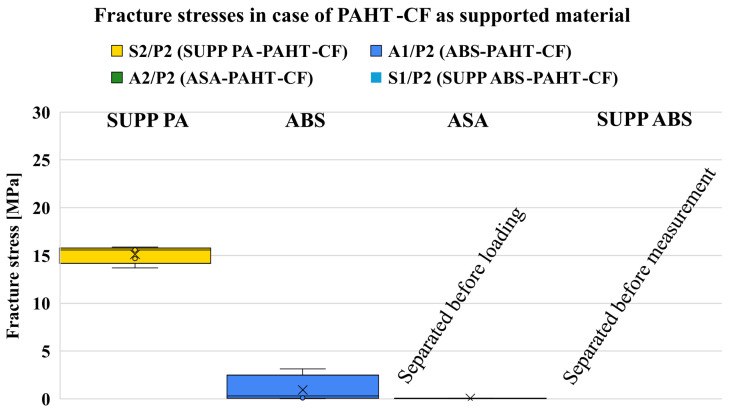
Fracture stresses of different combinations when supporting PAHT-CF.

**Figure 15:**
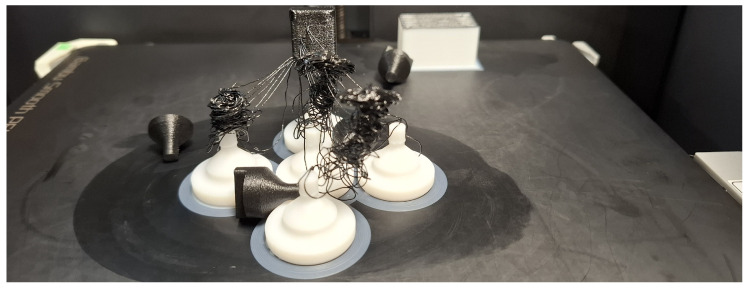
Printing failure of PAHT-CF on SUPP ABS support during manufacturing.

**Figure A4:**
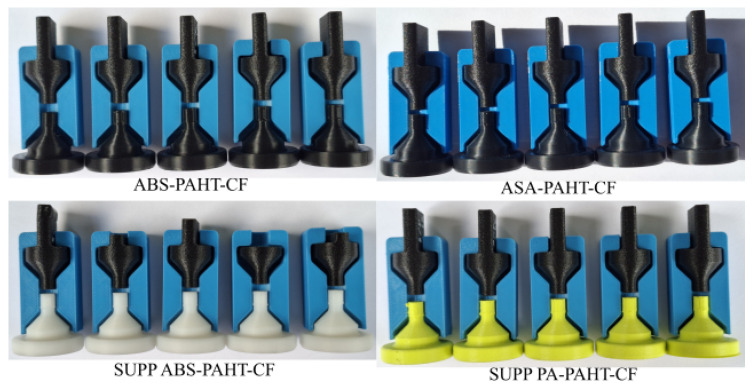
Fracture locations for PAHT-CF model material.

**Figure A5:**
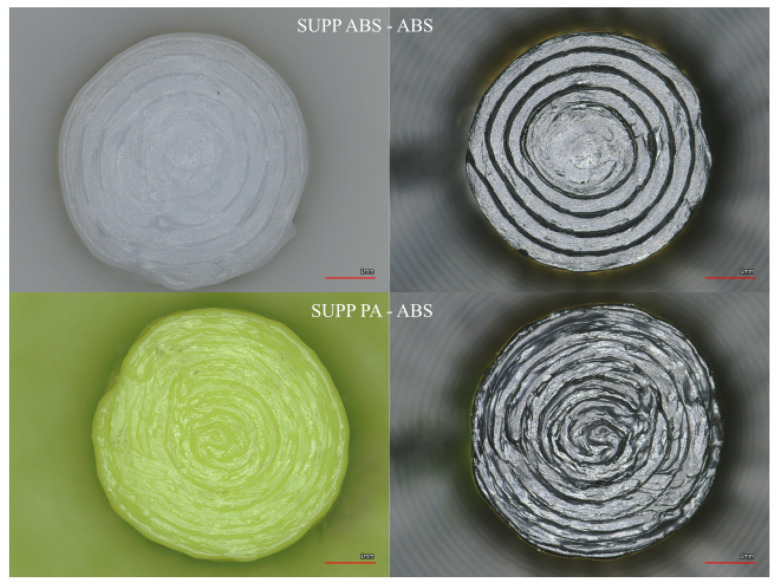
Fracture surfaces of ABS model material with SUPP ABS and SUPP PA supports.

**Figure A6:**
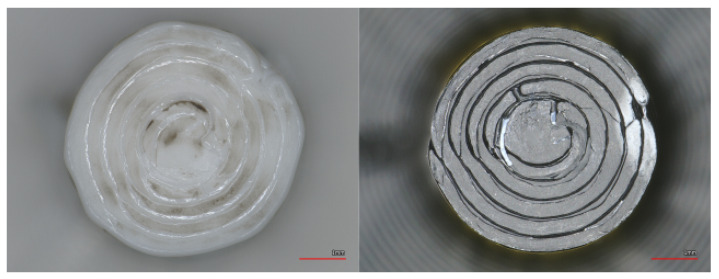
Fracture surface at the interface between PETG and ABS.

**Figure A7:**
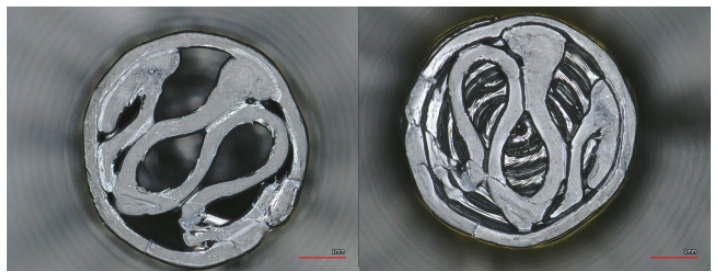
Fracture surface within the support structure between ASA and ABS.

**Figure A9:**
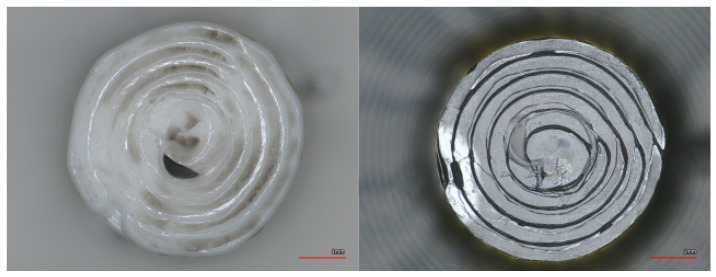
Diffusion observed at the interface between PETG and ASA.

**Figure A10:**
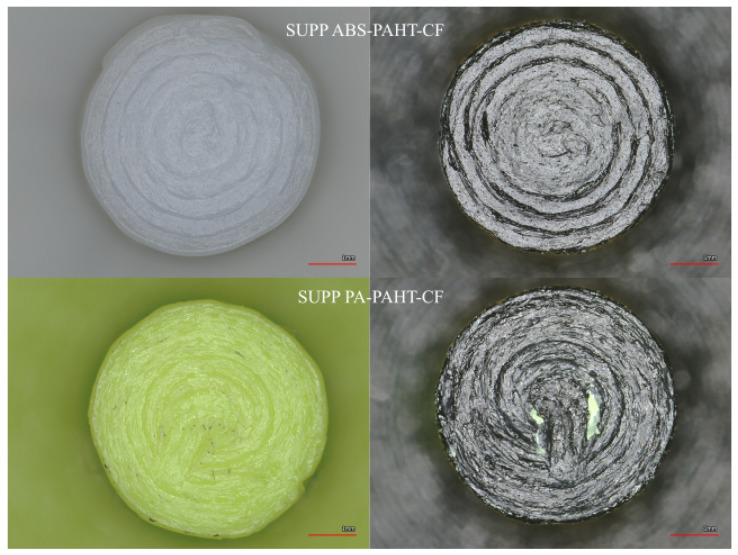
Fracture surfaces for PAHT-CF model material with SUPP ABS and SUPP PA supports.

**Figure A11:**
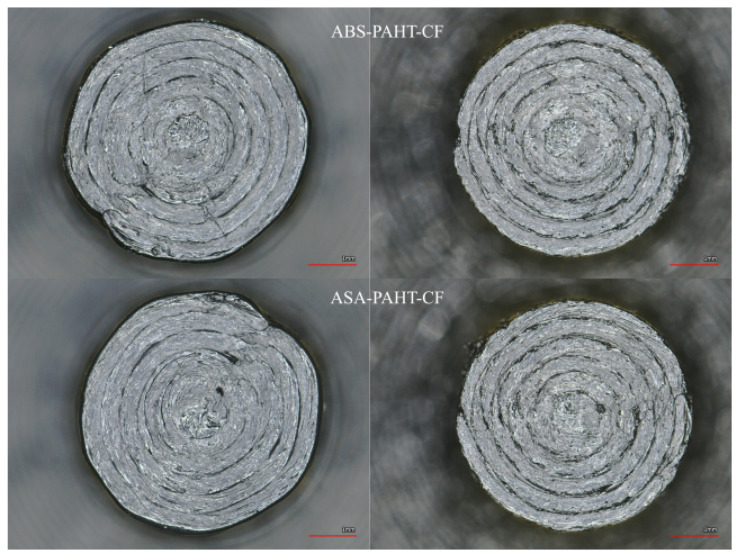
Fracture surfaces for PAHT-CF model material with ABS and ASA supports.

For PAHT-CF, somewhat unexpectedly, the dedicated support material SUPP PA produced the highest adhesion, which may appear surprising at first glance. However, inspection of Figure 14 reveals that for the other combinations, no measurable or only minimal adhesion was present. It can be caused by the carbon fiber blocking the diffusion and lowering the wetting [16]. Additionally, according to the literature, carbon fiber filling can accelerate cooling, leaving less time for chain diffusion and fusion of layers [47]. This can be problematic, as such low adhesion levels require careful consideration of whether the printed model is in contact with the build plate. If the part is built exclusively on support structures, the print head can easily displace it. A good example is the combination with SUPP ABS shown in Figure A4: only one out of five specimens remained on the support during printing, while the others detached and fell off (Figure 15). Nevertheless, examination of the fracture surfaces shows that, for SUPP PA, small fragments detached from the supported surface due to mechanical adhesion (Figure A10). This can cause issues for larger supported surfaces. SUPP ABS, on the other hand, produces a high-quality supported surface; however, as mentioned, it cannot be used as a stand-alone support for PAHT-CF and is only applicable when a substantial portion of the model is directly supported by the build plate. In the combinations with ABS and ASA shown in Figure A11, ASA provides a good-quality supported surface, yet adhesion remains negligible. ABS yields a similarly good supported surface, but with higher adhesion and consistent interfacial fracture without material remnants breaking off, making it the most suitable support material for PAHT-CF among the investigated combinations. The averaged measurement results are summarized in Table 7.

### 7.2. Evaluation of Surface Quality

**Figure 16:**
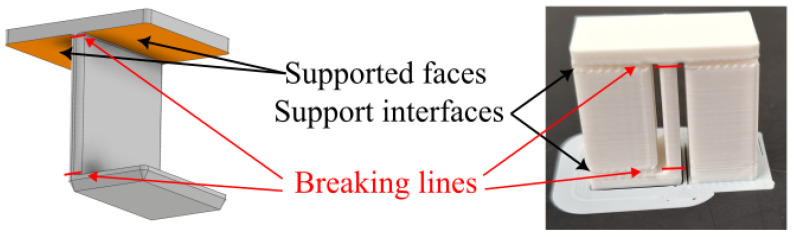
Breakable test part for observing the interfaces.

In addition to the quantitative evaluation, the combinations considered suitable were reprinted using dedicated test parts designed specifically for assessing support performance [48]. The specimen geometry simulates both enclosed support structures (typical in the support of horizontal holes) and support elements built from the build plate. After removing the supports, the specimens were fractured to expose the supported surfaces for microscopic documentation (see Figure 16). Both the supporting surfaces and the upper shell layers to which the supports were attached were examined and documented.

#### 7.2.1. Surface Quality of the ABS and SUPP ABS Combination

**Figure A12:**
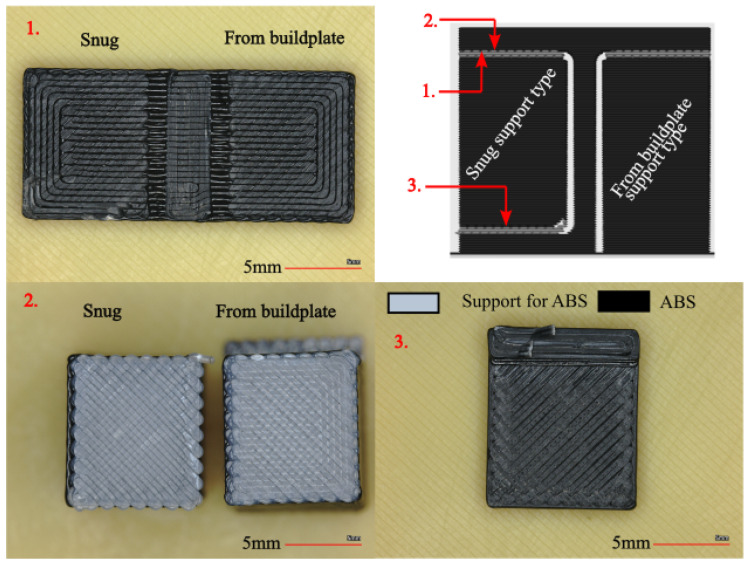
ABS surfaces supported by SUPP ABS.

The ABS and SUPP ABS combination produced supports that were easy to remove and surfaces with few defects. The snug-fitting intermediate support structure could be easily pulled out from between the model layers. The inclined pattern of the contacting support layer was imprinted on the supported surface, but this only resulted in a surface waviness on the order of a few hundredths of a millimeter. The ABS was able to spread well on the support, and only minor extrusion defects were visible, which also appeared on the third reference surface and thus cannot be attributed to the support itself (Figure A12). It can also be observed that material accumulation occurred at the edges of the contacting support layers.

#### 7.2.2. Surface Quality of the ABS and SUPP PA Combination

**Figure A13:**
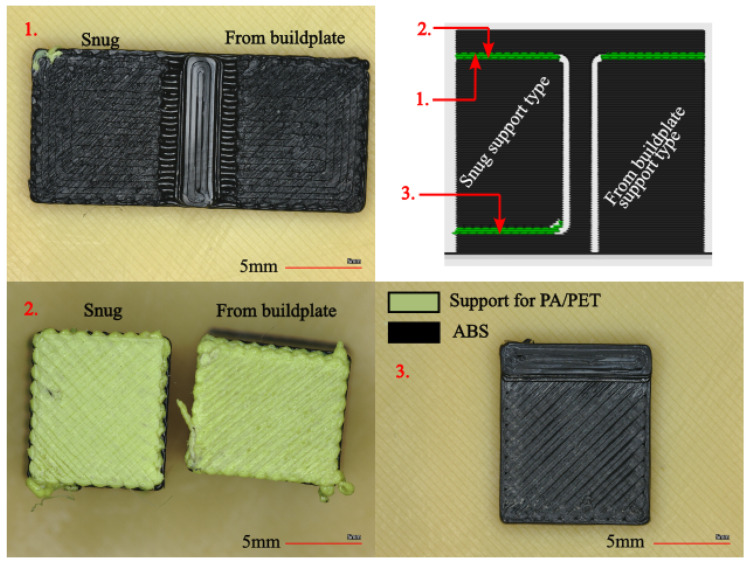
ABS surfaces supported by SUPP PA.

For SUPP PA, the resulting surface quality is similar in character to that obtained with SUPP ABS; however, support removal is more difficult, as also reflected by the higher fracture stresses. The support could not be removed by hand alone; a blade was required to separate the contacting layer. In Figure A13, residual support material fully fused with the model can still be seen. Material accumulation at the edges and significant stringing also appear on the part.

#### 7.2.3. Surface Quality of the ASA and SUPP ABS Combination

In this case, support removal could be performed manually; however, the contacting support layer detached not only from the model but also from the support structure during post-processing. The surface of the contacting layer shows strong similarity to that observed in the ABS case. The supported surfaces indicate that the material spread adequately and that proper support was achieved without major defects.

#### 7.2.4. Surface Quality of the ASA and SUPP PA Combination

Since the material structure of ASA is similar to that of ABS, a similar behavior was expected when using SUPP PA as support. The stringing and extrusion defects observed in the contacting layer again appear, and small amounts of support material remain adhered to the supported surface, which could only be removed with a blade at the risk of damaging the model.

#### 7.2.5. Surface Quality of the PAHT-CF and SUPP ABS Combination

**Figure A14:**
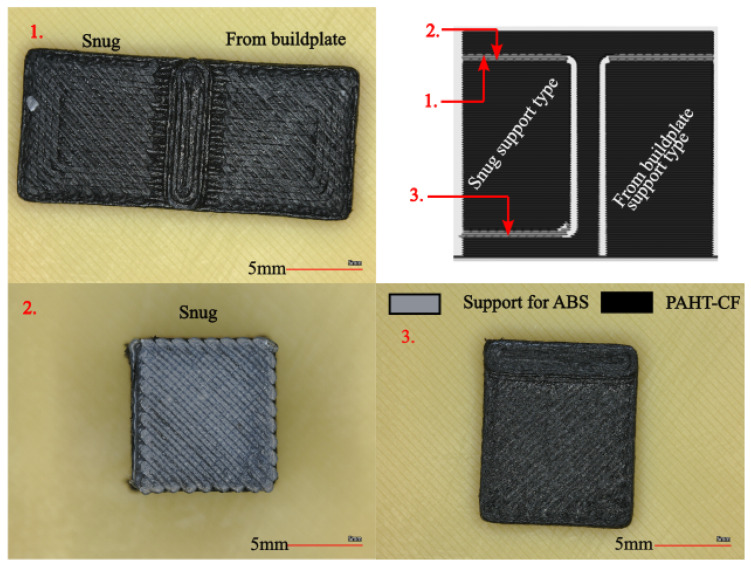
PAHT-CF surfaces supported by SUPP ABS.

In the PAHT-CF and SUPP ABS combination, adhesion was so low that the intermediate PA support structure could be removed by hand, and the contacting layers could then be peeled off the surface as a thin film. The quality of the supported surface is noticeably better than that observed on the tensile test specimens, most likely due to the infill pattern of the contacting layer (Figure A14). For carbon-fiber-reinforced composite filaments, a smooth surface is not typically expected; the rough, matte appearance is characteristic of such materials due to the fibers.

#### 7.2.6. Surface Quality of the PAHT-CF and SUPP PA Combination

**Figure 17:**
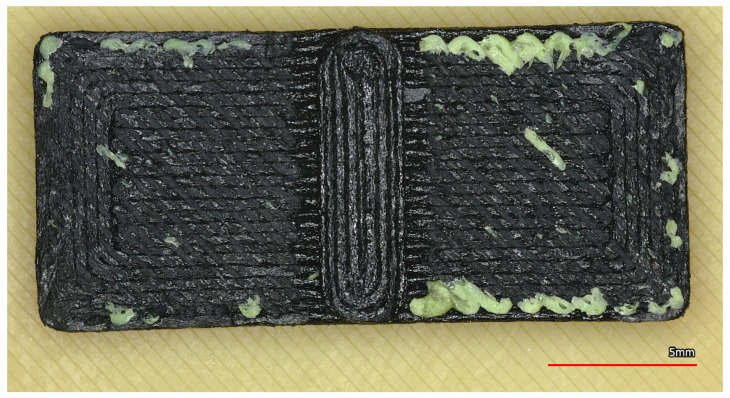
PAHT-CF surfaces supported by SUPP PA.

Testing this combination clearly shows that, although SUPP PA is the support material recommended for PAHT-CF, a gap should be introduced between support and model surfaces, as the adhesion is otherwise too strong. The surface shown in Figure 17 was obtained after separating the contacting support layer with a blade, yet a substantial amount of support material remained adhered to the model. Removing these residues would inevitably lead to damage to the part.

## 8. Conclusions

Following the increasing trend of multi-material FFF 3D printers, an attempt was made to investigate one of the most critical aspects of the technology: the interface between the model and the support structure. The literature review revealed that no unambiguous assessment is available regarding which support materials provide the best surface quality and, at the same time, adequate adhesion for a given model material. To address this gap, a six-component material set was examined in a combinatorial experimental campaign. During the work, it became evident that the problem is complex, as the requirements imposed on the support depend on numerous parameters, the investigation of which demands careful planning and documentation. In this work, the workflow started from the development of the measurement methodology to functional tests regarding polymer adhesion. Based on the obtained results, several pieces of information and data were generated that, to the author’s knowledge, are not yet available in the literature, thereby contributing to the development and better understanding of this segment of additive manufacturing.

**Figure 18:**
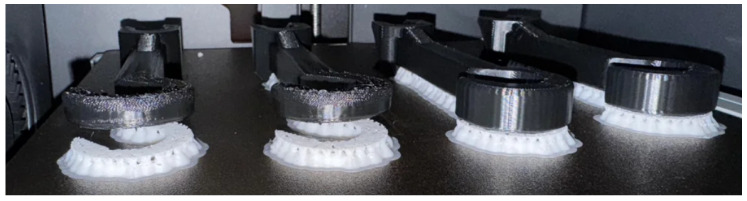
Warping of ABS on a SUPP ABS support structure due to inadequate chamber temperature [49].

For the investigation of adhesion between different materials, conventional flat dog-bone specimens were found to be unsuitable, and existing standards do not specifically address the testing of multi-material parts. For this reason, a custom specimen geometry and matching fixture were developed, and dedicated tensile tests were performed. These were found to be appropriate for comparative evaluation of the material combinations, and the measured scatter of the results remained below the scatter in tensile strength values reported in material data sheets. During manufacturing and testing, it became clear that the desired adhesion between model and support should neither be zero nor excessively high: the bond should remain intact for the duration of printing, but no longer. Based on the results, the dedicated support materials indeed provided lower adhesion overall; however, in the case of SUPP PA, higher adhesion was observed precisely with the two materials for which it is recommended (PAHT-CF and PETG). Overall, SUPP ABS proved to be a more versatile and effective support material for several model materials. Another important observation was that, when supporting PAHT-CF, ABS and ASA performed better as supports than SUPP PA. Combinations with no or only minimal adhesion were also identified. These pairs are already separated during manufacturing, making their practical applicability questionable. In applications where the model is in contact with the build plate and only partially requires support, such material pairs may be perfectly adequate; however, if the entire part is supported, the print head is likely to dislodge the component. Warpage tendency represents an additional challenge, as the support must also counteract deformations arising from thermal stresses (a striking example of this is shown in Figure 18). In future work, particular attention should be paid to identifying the optimal environmental temperatures for combinations with significantly different coefficients of thermal expansion, in order to prevent separation during printing.

To further validate the tensile test results, additional specimen types and testing methods are required. In the tensile specimen geometries found in the literature, the inclusion of a gap that would prompt the slicer to generate a support structure between the materials was not observed. In view of this, the existing solutions are not considered to fully reflect practical usage, since at the material transition, the slicer always generates toolpaths with significantly different process parameters. In addition to modifying the geometry, it is also necessary to investigate other loading modes. In the present study, the specimens were subjected to purely tensile loading; in practice, however, support removal corresponds to a peeling-type load at the interface. A peel test would therefore be more suitable to describe the actual interfacial behavior between the materials. With regard to process parameters, it was demonstrated that the printer produces the first model layer at a much higher speed and with stronger cooling than the subsequent layers. During the design of the experiments and slicing, this behavior was an inherent default setting in Bambu Studio version 1.10 and was intentionally left unchanged. By the time this work approached completion, version 2.0 of the software was released, in which, again using default settings, this speed relationship is reversed: the first deposited layer is printed much more slowly. This raises questions regarding the manufacturer’s rationale. Although no response was received from the company to an inquiry on this topic, such a fundamental change clearly warrants further investigation, and tensile test results obtained with the new toolpaths should be compared with those presented here.

The most promising material pairings, selected based on the tensile stress results, were also produced using a specimen type with a larger supported surface area. The corresponding surface images indicate that, in some cases, deviations from the default settings would be required in order to further improve surface quality; however, such modifications could not be implemented within the available time frame. Nevertheless, the flattened and smoothed lower surfaces showed significantly higher quality than those obtained with a deliberate gap between model and support. This was considered a step forward in improving the efficiency and usability of the technology. In future work, systematic variation in volumetric flow rate and temperature should be carried out to identify parameter sets that provide further surface quality improvements and to determine the optimal process window.

On the basis of the presented results, several directions for further development and research needs have emerged. One such direction arises from the fact that, during the course of this work, Bambu Lab extended its material portfolio with several new model materials; inclusion of these in the combination matrix and their subsequent testing would yield additional, highly valuable data. Moreover, it would be beneficial for a dedicated testing methodology and specimen geometry to be developed that would contribute as effectively as possible to the characterization of interfacial adhesion between polymers. The lack of suitable standards, combined with the increasingly widespread adoption of multi-material printing, leads to processes that are less and less regulated. Since standardization is unable to keep pace with the development of this branch of technology, the comparability of research data may become questionable in some cases in the absence of harmonization. There is, therefore, a need for test methods, specimen geometries, and prescribed parameter sets to be defined that can be implemented both on consumer-grade and industrial machines, ensuring that results are reproducible and comparable across different studies and hardware setups.

## Figures and Tables

**Table 1:** Nozzle temperatures of the investigated materials according to datasheets and slicer profiles [32,33,34,35,36,37].

ID	Name	Datasheet Nozzle Temp. [°C]	Slicer Profile Nozzle Temp. [°C]
A1	ABS	240–270	270
A2	ASA	240–270	270
P1	PETG	240–270	255
P2	PAHT-CF	260–290	290
S1	SUPP ABS	240–270	270
S2	SUPP PA	260–290	280

**Table 2:** Absolute differences between printing temperatures of base materials [32,33,34,35,36,37]. The combinations with over 25 °C difference are highlighted in red.

ID	Name	ABS	ASA	PETG	PAHT-CF	SUPP ABS	SUPP PA
A1	ABS	0	0	15	20	0	10
A2	ASA	0	0	15	20	0	10
P1	PETG	15	15	0	35	15	25
P2	PAHT-CF	20	20	35	0	20	10
S1	SUPP ABS	0	0	15	20	–	–
S2	SUPP PA	10	10	25	10	–	–

**Table 3:** Individually adjusted parameters in the 0.2 mm default Bambu profile (version 1.7.1.62).

Parameter	Value
Layer height	0.2 mm
Seam position	Back
Wall loops	6
Top surface pattern	Monotonic
Support style	Snug
Top Z distance	0 mm
Bottom Z distance	0 mm
Base pattern	Honeycomb
Base pattern spacing	1 mm
Top interface layers	3 layers
Bottom interface layers	3 layers
Interface pattern	Rectilinear interlaced
Top interface spacing	0 mm

**Table 4:** Averaged measurement results for materials supporting ABS.

Material	*D*_fracture_ [mm]	*F*_max_ [N]	*σ*_max_ [MPa]
ASA	4.84	350.09	19.05 ± 0.93 (n = 5)
PETG	4.85	425.30	22.98 ± 1.21 (n = 4)
SUPP ABS	4.85	115.57	6.26 ± 3.1 (n = 5)
SUPP PA	4.76	152.71	8.58 ± 1.37 (n = 5)

**Table 5:** Averaged measurement results for materials supporting ASA.

Material	*D*_fracture_ [mm]	*F*_max_ [N]	*σ*_max_ [MPa]
ABS	4.83	344.23	18.81 ± 1.17 (n = 3)
PETG	4.81	344.90	18.99 ± 0.82 (n = 3)
SUPP ABS	4.80	72.40	4.00 ± 0.56 (n = 5)
SUPP PA	4.74	122.62	6.94 ± 1.2 (n = 5)

**Table 6:** Averaged measurement results for materials supporting PETG.

Material	*D*_fracture_ [mm]	*F*_max_ [N]	*σ*_max_ [MPa]
ABS	4.83	245.97	13.40 ± 4.32 (n = 5)
ASA	4.73	160.86	9.15 ± 4.78 (n = 3)
SUPP ABS	4.76	82.21	4.62 ± 1.13 (n = 4)
SUPP PA	4.67	177.04	10.33 ± 0.65 (n = 5)

**Table 7:** Averaged measurement results for materials supporting PAHT-CF.

Material	*D*_fracture_ [mm]	*F*_max_ [N]	*σ*_max_ [MPa]
ABS	4.86	17.37	0.94 ± 0.94 (n = 4)
ASA	4.89	0.92	0.05 ± 0.05 (n = 2)
SUPP ABS	No data	No data	No data
SUPP PA	4.79	271.93	15.11 ± 0.82 (n = 5)

**Figure A8:**
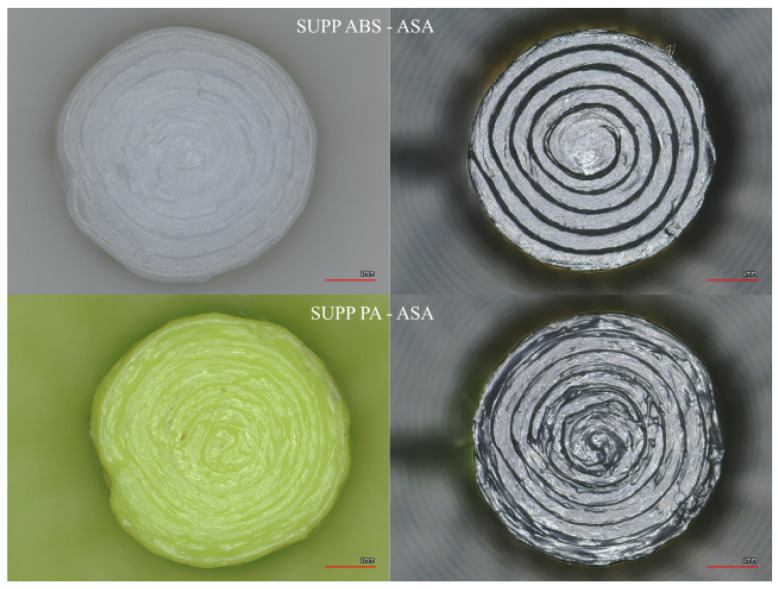
Fracture surfaces of ASA model material supported by SUPP ABS and SUPP PA.

**Figure A15:**
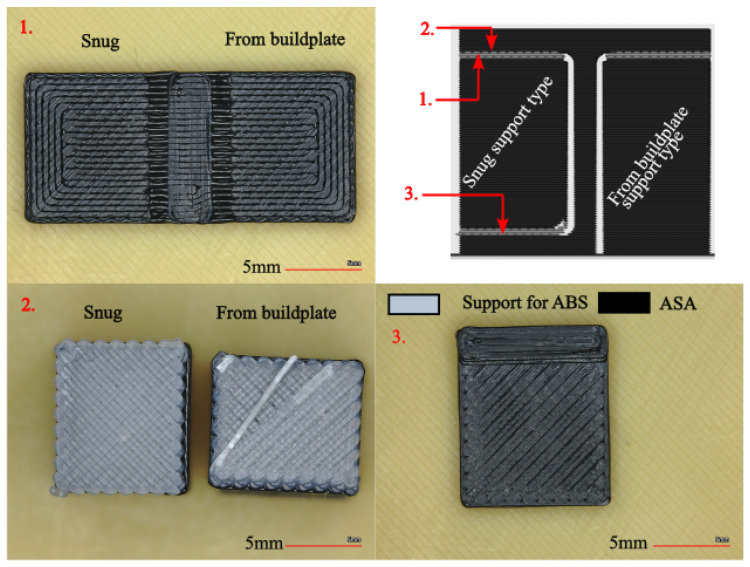
ASA surfaces supported by SUPP ABS.

**Figure A16:**
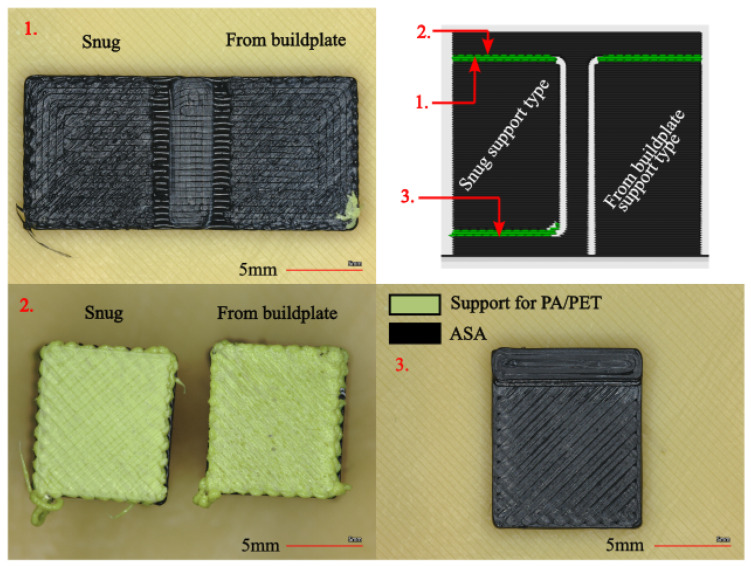
ASA surfaces supported by SUPP PA.

## Data Availability

The raw data supporting the conclusions of this article will be made available by the authors on request.

## Abbreviations

The following abbreviations are used in this manuscript:

FDM	Fused Deposition Modelling
FFF	Fused Filament Fabrication
ABS	Acrylonitrile Butadiene Styrene
ASA	Acrylonitrile Styrene Acrylate
PETG	Polyethylene Terephthalate Glycol
PAHT	Polyamide 12 High Temperature
DfAM	Design for Additive Manufacturing
HIPS	High Impact Polystyrene

## Appendix A

**Table A1:** Relevant physical and thermal properties of the the measured materials according to their datasheets [32,33,34,35,36,37].

Material Name	Tensile Strength [MPa]	Melting Temperature [°C]
ABS	28 ± 2	200
ASA	31 ± 4	210
PETG	35 ± 6	No data
PAHT-CF	47 ± 5	225
SUPP ABS	No data	195
SUPP PA	No data	255
